# Hermansky-Pudlak syndrome and oculocutaneous albinism in Chinese children with pigmentation defects and easy bruising

**DOI:** 10.1186/s13023-019-1023-7

**Published:** 2019-02-21

**Authors:** Bradley Power, Carlos R. Ferreira, Dong Chen, Wadih M. Zein, Kevin J. O’Brien, Wendy J. Introne, Joshi Stephen, William A. Gahl, Marjan Huizing, May Christine V. Malicdan, David R. Adams, Bernadette R. Gochuico

**Affiliations:** 1 Medical Genetics Branch, National Human Genome Research Institute, National Institutes of Health, 10 Center Drive, MSC 1851, Bethesda, MD 20892-1851 USA; 2 Division of Hematopathology, Department of Laboratory Medicine and Pathology, Mayo Clinic, Rochester, MN USA; 3 National Eye Institute, National Institutes of Health, Bethesda, MD USA; 4 Office of the Clinical Director, National Human Genome Research Institute, National Institutes of Health, Bethesda, MD USA; 5 Undiagnosed Diseases Program, NIH Common Fund, Office of the Director, National Institutes of Health, Bethesda, MD USA

**Keywords:** Bruising, Chinese, Dense granule, Hermansky-Pudlak syndrome, Hypopigmentation, Oculocutaneous albinism, Platelet

## Abstract

**Background:**

Determining the etiology of oculocutaneous albinism is important for proper clinical management and to determine prognosis. The purpose of this study was to genotype and phenotype eight adopted Chinese children who presented with oculocutaneous albinism and easy bruisability.

**Results:**

The patients were evaluated at a single center; their ages ranged from 3 to 8 years. Whole exome or direct sequencing showed that two of the children had Hermansky-Pudlak syndrome (HPS) type-1 (HPS-1), one had HPS-3, one had HPS-4, and four had non-syndromic oculocutaneous albinism associated with *TYR* variants (OCA1). Two frameshift variants in *HPS1* (c.9delC and c.1477delA)*,* one nonsense in *HPS4* (c.416G > A), and one missense variant in *TYR* (c.1235C > T) were unreported. The child with HPS-4 is the first case with this subtype reported in the Chinese population. Hypopigmentation in patients with HPS was mild compared to that in OCA1 cases, who had severe pigment defects. Bruises, which may be more visible in patients with hypopigmentation, were found in all cases with either HPS or OCA1. Whole mount transmission electron microscopy demonstrated absent platelet dense granules in the HPS cases; up to 1.9 mean dense granules per platelet were found in those with OCA1. Platelet aggregation studies in OCA1 cases were inconclusive.

**Conclusions:**

Clinical manifestations of oculocutaneous albinism and easy bruisability may be observed in children with HPS or OCA1. Establishing definitive diagnoses in children presenting with these phenotypic features is facilitated by genetic testing. Non-syndromic oculocutaneous albinism and various HPS subtypes, including HPS-4, are found in children of Chinese ancestry.

## Background

Hypopigmentation of the skin, hair and eyes is a manifestation of various inherited disorders, including non-syndromic oculocutaneous albinism (OCA), Hermansky-Pudlak syndrome (HPS), Chediak-Higashi syndrome, and Griscelli syndrome [1–3]. The underlying cellular mechanisms causing pigmentation defects differ in each of these disorders. Non-syndromic OCA (MIM #203100), comprised of 7 genetically distinct disorders, is characterized by hypopigmentation, due to absent or reduced melanin biosynthesis within melanocytes [4, 5]. There are 10 known genetic subtypes of HPS (MIM #203300, #614072, #614073), characterized by hypopigmentation and a bleeding diathesis, associated with abnormal biogenesis of lysosome-related organelles, which include melanosomes that are melanin-containing organelles within melanocytes [3]. Chediak-Higashi syndrome (MIM#214500) is a rare lysosomal disorder associated with biallelic variants in *LYST* and enlarged, membrane-rich, lysosome-related organelles, resulting in pigmentary, hematologic, and neurologic involvement [6, 7]. Griscelli syndrome (MIM#214450), characterized by hypopigmentation, is a melanosome transport disorder associated with variants in *MYO5A*, *RAB27A*, or *MLPH* [2]. Pigmentation is generally reduced in these disorders, but the severity of the pigment defect is variable.

Non-syndromic OCA and HPS are autosomal recessive disorders that share some, but not all, clinical features. Manifestations of both non-syndromic OCA and HPS include decreased skin and hair pigmentation, nystagmus, iris transillumination, reduced visual acuity, increased optic nerve decussation, and foveal hypoplasia [1–3, 8]. HPS, but not non-syndromic OCA, is further characterized by a bleeding diathesis due to a platelet storage pool defect [9]. In addition, some patients with HPS develop granulomatous colitis, neutropenia, or pulmonary fibrosis [2, 3, 8]. Specifically, children and young adults with HPS type-2 (HPS-2) can develop neutropenia responsive to granulocyte-stimulating factor, immunodeficiency, interstitial lung disease, and/or pulmonary fibrosis [10–13]. Fibrotic lung disease is highly prevalent in middle-aged adults with HPS-1 and HPS-4, and this condition is associated with substantial morbidity and mortality [9, 14, 15]. Given the high risk of fibrotic lung disease in patients with some subtypes of HPS, but not in non-syndromic OCA, establishing an accurate diagnosis of HPS in patients with oculocutaneous albinism and identifying their HPS subtype are essential for anticipating symptoms, providing proper clinical management, and for estimating prognosis.

The prevalence of non-syndromic OCA and HPS differs considerably. The worldwide prevalence of all known forms of OCA is estimated to be 1 per 17,000 [16]. OCA1, which is associated with variants in *TYR*, affects approximately 1 per 40,000 in the general population [17, 18]. OCA is reported to affect approximately 1 per 18,000 in the Chinese Han population in the eastern Chinese province of Shandong [5]. In contrast, HPS is estimated to occur in only 1–9 persons per 1,000,000 worldwide and, similar to OCA, also varies based on genetic subtype and region. For example, Puerto-Ricans are predisposed to HPS-1 and HPS-3 due to the presence of founder variants in the *HPS1* and *HPS3* genes in the Puerto-Rican population [19, 20]. HPS-1 affects 1 per 1800 persons in northwest Puerto Rico, and HPS-3 occurs in 1 per 16,000 persons throughout Puerto Rico [3, 20, 21]. Thus far, few patients of Chinese ancestry are reported with HPS [22–25].

In general, ocular findings and hypopigmentation are prominent features of patients affected with either HPS or non-syndromic OCA, and patients can be misdiagnosed [21]. HPS is rare, so children with oculocutaneous albinism are initially suspected of having non-syndromic OCA. Conversely, although excessive bruising is a feature of HPS, bruises in patients with non-syndromic OCA can be readily apparent due to reduced skin pigmentation, and ecchymoses in a person with albinism may be interpreted as easy bruisability. Hence, some children with non-syndromic OCA and a normal amount of bruising may be initially misdiagnosed with HPS.

We report eight adopted children of Chinese ancestry with oculocutaneous albinism, a tendency to bleed, and platelet dysfunction who were suspected to have HPS. Whole exome or direct sequencing analyses revealed variants in genes associated with HPS or the tyrosinase gene (*TYR*). Two children were diagnosed with HPS-1, one with HPS-3, one with HPS-4, and four with OCA1. Children with HPS had absent dense granules; those with OCA1 had low numbers of dense granules per platelet and inconclusive platelet functional test results. Some of their genetic variants are novel, and the patient with HPS-4 is the first reported HPS-4 case of Chinese ethnicity. This study highlights the importance of genetic testing in establishing an accurate diagnosis in patients of Chinese ancestry with albinism and suspected bleeding diathesis.

## Results

### Patient genotyping

Whole exome or Sanger sequencing analysis identified homozygous or compound heterozygous variants in *HPS1* (NM_000195.4), *HPS3* (NM_032383.4), *HPS4* (NM_022081.5), or *TYR* (NM_000372.4) in 8 children of Chinese descent (Table 1). Single-nucleotide polymorphism analysis excluded hemizygosity in the child (435) with a homozygous variant in *HPS4*, but hemizygosity is possible in the two children (215 and 351) whose variants were identified by direct sequencing. No pathogenic variants were identified in genes associated with Chediak-Higashi syndrome (*LYST*) or Griscelli syndrome (*MYO5A*, *RAB27A*, *MLPH*) in the 6 patients who underwent whole exome sequencing analysis. None of the children were known to be related, but three children with OCA1 were from the same orphanage. Four patients originated from the Nanjing area, one from Beijing, one from northwest China, one from southern China, and one from an unknown region of China (Table 2). Limited to no information was available about the children’s biological parents, and thus, history of consanguinity is unknown. Two of four children with HPS had HPS-1. One patient (215) had a frameshift variant in *HPS1*, and the other patient (471) had compound heterozygous frameshift variants, leading to premature termination codons. Two of the three *HPS1* variants (c.9delC; p.Cys3Trpfs*26 and c.1477delA; p.Arg493Glyfs*22) were not previously reported. The third variant (c.972dupC; p.Met325Hisfs*128) is a frequently reported *HPS1* variant in cases of different ethnicities, including Caucasian and Chinese [23, 26, 27]. One child (351) had a variant in *HPS3* (c.1555_1595dup41; p.Leu533Phefs*10). A query of the ExAC and GnomAD databases showed that the allele frequency for this mutation is very low in East Asian populations, suggesting that this variant is rare. One patient (435) with HPS-4 was homozygous for a novel nonsense variant in *HPS4* (c.416G > A; p.Trp139*); this variant is not reported in ExAc nor GnomAD databases.

**Table 1:** Gene Variants in our Cohort and Frequencies in the East Asian Population

Patient	Gene	Variant 1	East Asian MAF^a^	Variant 2	East Asian MAF^a^
215	*HPS1*	c.9delC p.Cys3Trpfs*26	N/R	c.9delC p.Cys3Trpfs*26	N/R
351	*HPS3*	c.1555_1595dup p.Leu533Phefs*10	0.0001160 (gnomAD)	c.1555_1595dup p.Leu533Phefs*10	0.0001160 (gnomAD)
435	*HPS4*	c.416G > A p.Trp139*	N/R	c.416G > A p.Trp139*	N/R
471	*HPS1*	c.972dupC p.Met325Hisfs*128	0.001458 (ExAc) ^b^	c.1477delA p.Arg493Glyfs*22	N/R
468	*TYR*	c.929dup p.Arg311Lysfs*7	0.000694 (ExAc) ^c^	c.896G > A p.Arg299His	0.000347 (ExAc) ^d^
469	*TYR*	c.230_232dupGGG p.Arg77_Glu78insGly	0.0001168 (ExAc)	c.896G > A p.Arg299His	0.000347 (ExAc) ^d^
470	*TYR*	c.230_232dupGGG p.Arg77_Glu78insGly	0.0001168 (ExAc)	c.1235C > T; p.Pro412Leu	N/R
474	*TYR*	c.655G > A p.Glu219Lys	N/R ^e^	c.896G > A p.Arg299His	0.000347 (ExAc) ^d^

*MAF* Minor Allele Frequency, *N/R* None reported

^a^The ExAc (Beta; Exome Aggregation Consortium; http://exac.broadinstitute.org/) and GnomAD browsers (Beta; Genome Aggregation Database; http://gnomad.broadinstitute.org/)

were searched in September 2018

^b^ClinVar Accession RCV000005596.4

^c^ClinVar Accession RCV000003969.5

^d^ClinVar Accession RCV000004000.4

^e^Reported in Reference [29]; not reported in ExAc or GnomAD

**Table 2:** Patient Characteristics

Patient	Diagnosis	Region	Age (yrs)	Gender	Bruising	Epistaxis	Infections
215	HPS-1	Northwest	6	Male	yes	yes	no
351	HPS-3	South	6	Female	yes	yes	no
435	HPS-4	Nanjing	3	Male	yes	yes	no
471	HPS-1	N/A	3	Female	yes	no	no
468	OCA1	Nanjing	8	Female	yes	no	yes
469	OCA1	Nanjing	5	Male	yes	no	yes
470	OCA1	Nanjing	3	Female	yes	no	yes
474	OCA1	Beijing	8	Female	yes	yes	no

*HPS*, Hermansky-Pudlak syndrome

*N/A*, not available

*OCA*, oculocutaneous albinism

Four patients (468, 469, 470, 474) with compound heterozygous variants in *TYR* were diagnosed with OCA1. Three children (468, 469, 474) had one previously reported missense variant in common (c.896G > A; p.Arg299His), but their second variant was different. Overall, five variants in *TYR* were found in these four individuals with OCA1. Two of these variants were previously reported (c.896G > A; p.Arg299His and c.230_232dupGGG; p.Arg77_Glu78insGly), including frequently in subjects of Chinese and Taiwanese ethnicity [22, 28]. Pathogenicity of the missense variant c.896G > A was ascribed to its location in the copper-binding site of TYR, while pathogenicity of c.230_232dupGGG was ascribed to truncation of the TYR peptide [22, 25]. One missense *TYR* variant (c.655G > A; p.Glu219Lys) was not reported in the ExAC and GnomAD variant databases, but was published [29]. The c.655G > A variant is located in a copper-binding site of TYR; in silico pathogenicity analysis predicted severe pathogenicity to protein function, including ‘damaging’ (score 0) by SIFT (http://sift.jcvi.org/), ‘probably damaging’ (score 1) by PolyPhen-2 (http://genetics.bwh.harvard.edu/pph2/), and ‘disease’ (score 0.92 [94%]) by PMut (http://mmb.pcb.ub.es/PMut/). One novel missense *TYR* variant (c.1235C > T; p.Pro412Leu) was not reported in the ExAC and GnomAD variant databases nor in the albinism database (http://www.ifpcs.org/albinism/). The c.1235C > T variant also yielded severe in silico pathogenicity prediction scores, including ‘damaging’ (score 0) by SIFT, ‘probably damaging’ (score 1) by PolyPhen-2, and ‘disease’(score 0.81 [89%]) by pMut.

### Clinical characteristics

**Fig. 1:**
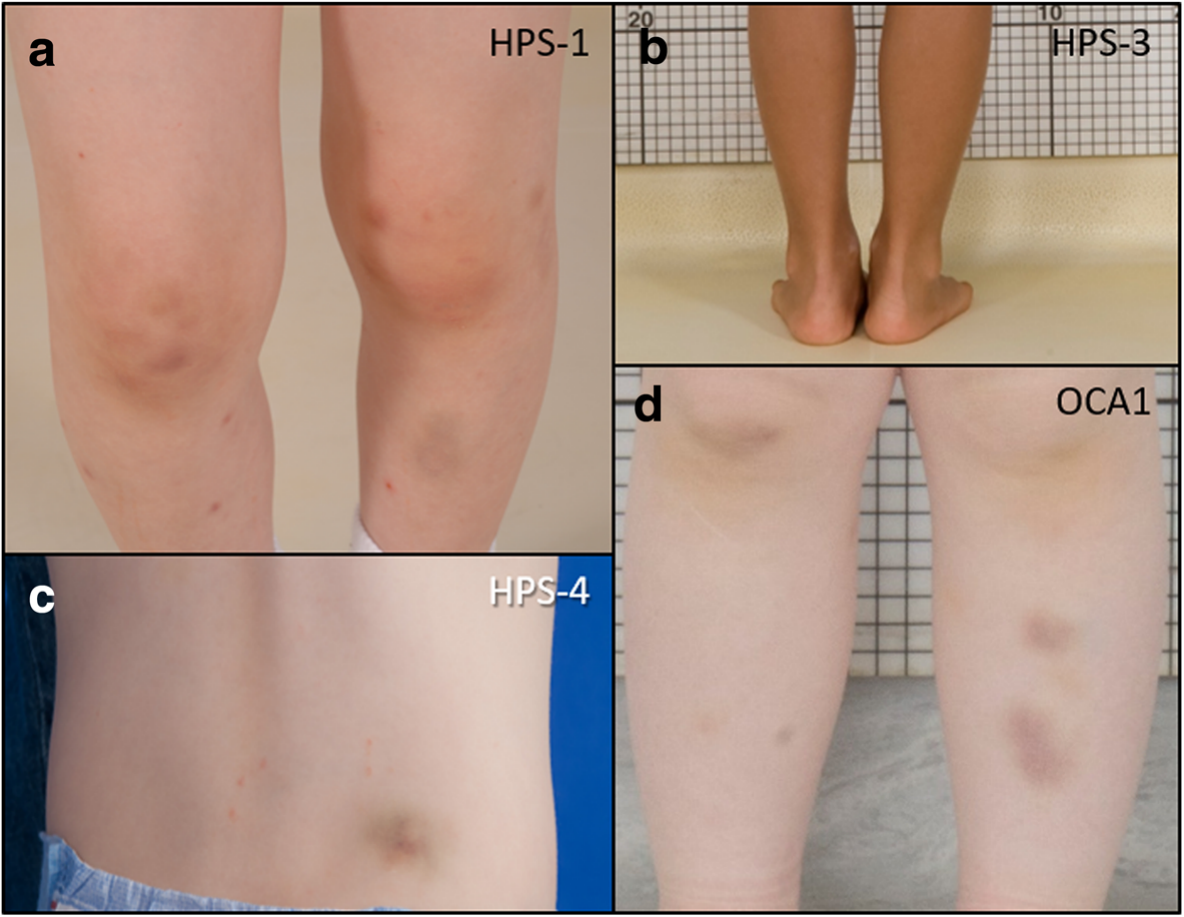
Bruising in Chinese Children with Hermansky-Pudlak Syndrome or Non-syndromic Oculocutaneous Albinism Type 1. Clinical images depict large or multiple bruises in different stages of resolution in four Chinese children with Hermansky-Pudlak syndrome (HPS) or non-syndromic oculocutaneous albinism type 1 (OCA1). Lower extremities of a patient with HPS-1 (**a**), HPS-3 (**b**), or OCA1 (**d**) are shown; posterior lumbar region of the patient with HPS-4 (**c**) is also shown. Variable degrees of skin pigmentation defects are evident in these four Chinese children. Hypopigmentation is milder in three children with HPS, especially the one with HPS-3, compared to one with OCA1, who has white skin

**Fig. 2:**
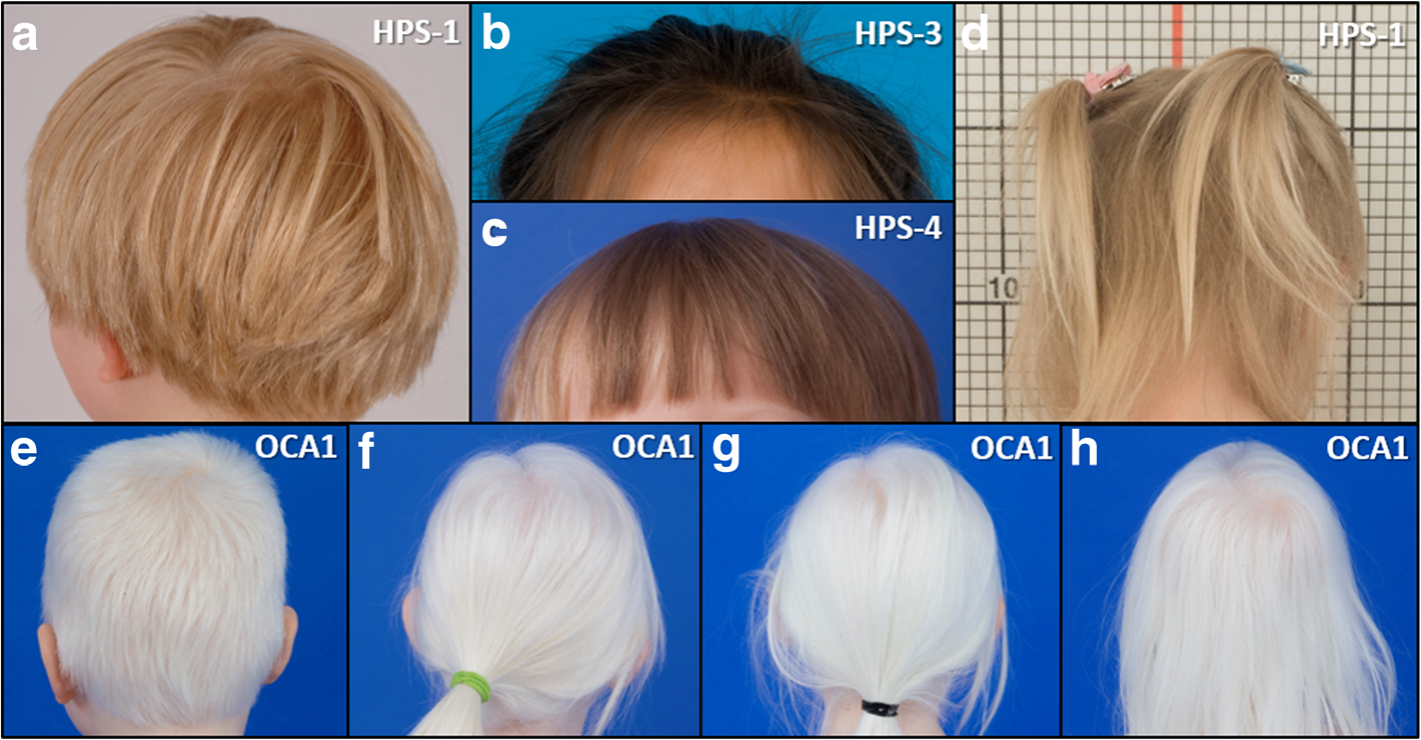
Hypopigmentation in Chinese Pediatric Patients with Hermansky-Pudlak Syndrome or Non-syndromic Oculocutaneous Albinism Type 1. An albinotic phenotype is observed in four Chinese children with Hermansky-Pudlak syndrome (HPS) (**a-d**) and four Chinese children with non-syndromic oculocutaneous albinism type 1 (OCA1) (**e-h**). The patients with HPS-1 or HPS-4 had light brown or blond hair (**a, c, or d**), which is atypical for Chinese individuals who generally have black hair. The child with HPS-3 had dark brown hair. A severe pigment defect is observed in four patients with OCA1, who had white hair (**e-h**)

**Fig. 3:**
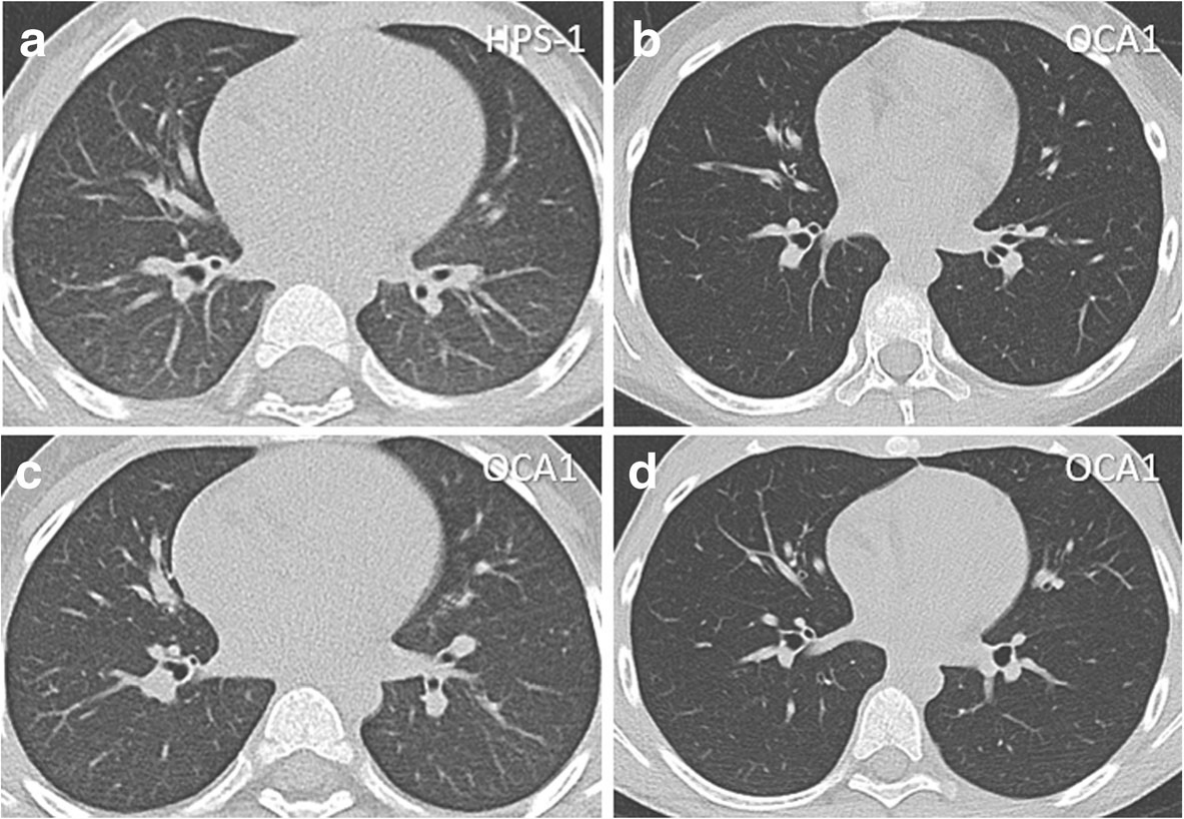
Chest Imaging in Chinese Children with Hermansky-Pudlak Syndrome or Non-syndromic Oculocutaneous Albinism Type 1. Representative images of computer tomography scans of the chest demonstrate normal lung parenchymal tissue in a Chinese pediatric patient with HPS-1 (**a**) or three with OCA1 (**b-d**). There is no radiographic evidence of interstitial lung disease or pulmonary fibrosis

The mean age of the patients with HPS was 4 years, 8 months and that of patients with OCA1 was 6 years, 2 months (Table 2). Two patients with HPS and one with OCA1 were male. The subjects initially presented with a mild albinotic phenotype and a tendency to bleed with multiple bruises at varying stages of resolution or in unusual sites (Fig. 1a-d). Patients with HPS-1 or HPS-4 had pale skin and light brown hair, which is atypical for individuals of East Asian ancestry who generally have black hair color (Fig. 2a-d). One child with HPS-3 had dark brown hair, and her skin was able to darken with exposure to sunlight. The patients with OCA1 generally had a more severe albinotic phenotype. These patients had white skin and hair indicating a severe pigment defect (Fig. 2e-h). All eight patients reported easy bruising (Table 2). Three children with HPS and one with OCA1 had epistaxis. Three children with OCA1 reported frequent upper respiratory tract infections with several episodes per year. No patients in either group reported colitis or lung disease, and chest computed tomography scans did not show interstitial lung disease or pulmonary fibrosis (Fig. 3a-d).

### Eye findings

**Fig. 4:**
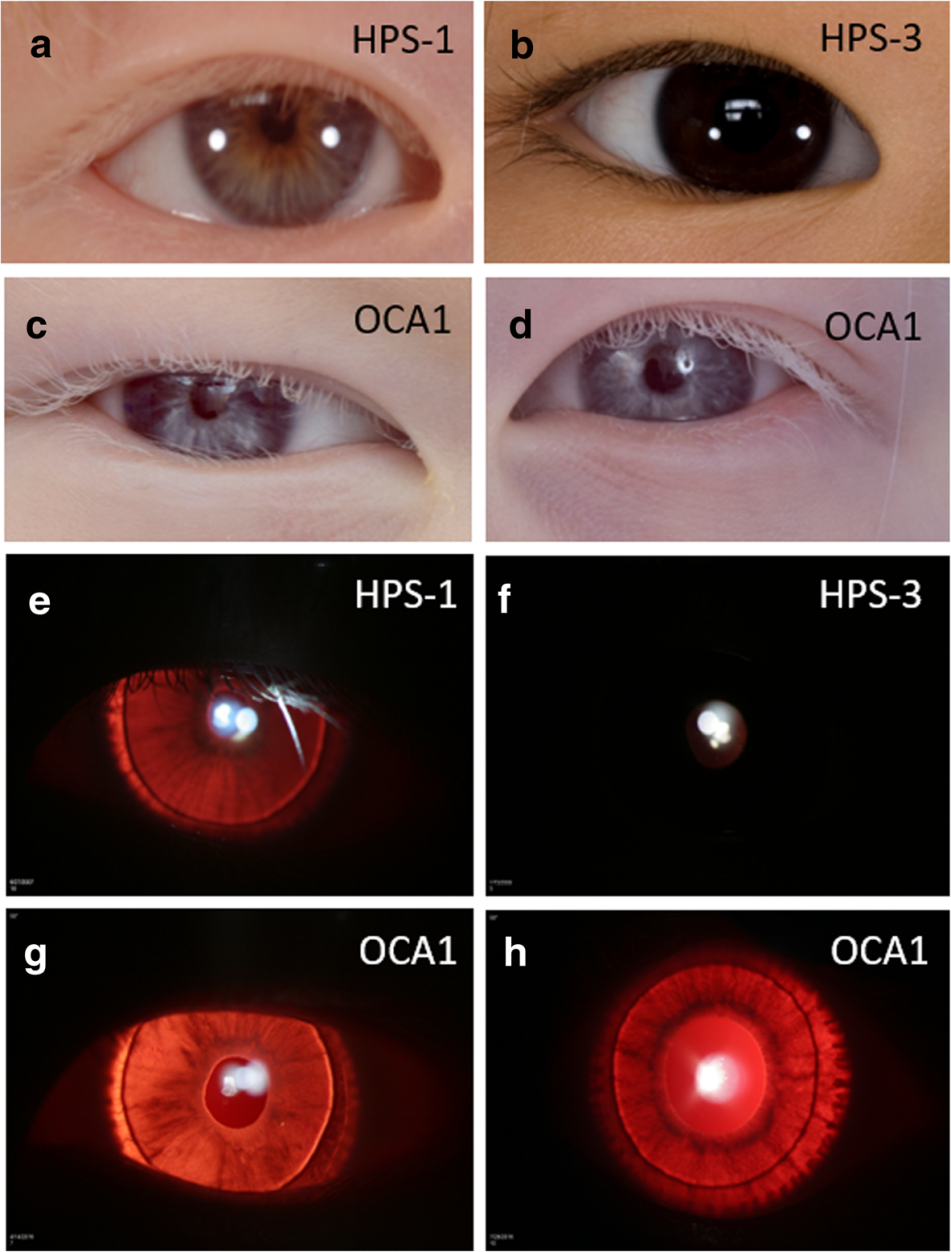
Ophthalmologic Findings in Chinese Pediatric Patients with Hermansky-Pudlak Syndrome or Non-syndromic Oculocutaneous Albinism Type 1. Representative images show light-colored eyes and eyelashes as well as iris transillumination in Chinese children with HPS-1 (**a**, **e**), HPS-3 (**b**, **f**), or OCA1 (**c**, **d**, **g**, **h**). Pigmentation defects in pediatric patients with HPS, particularly one with HPS-3 and dark brown irides, are not as severe as those in two patients with OCA1 and light blue irides. Eyelashes are blond in a child with HPS-1, dark brown in one with HPS-3, and white in two with OCA1

All subjects underwent ophthalmologic examination to assess visual acuity, nystagmus and motility, foveal hypoplasia, and iris transillumination (Table 3, Fig. 4a-h). Snellen visual acuity was abnormal in all patients. Visual acuity ranged from 20/80 to 20/400 in patients with HPS and 20/125 to 20/640 in those with OCA1. Although pigmentation defect was relatively mild in the child with HPS-3, visual acuity of this patient was similar to that of other cases with HPS or OCA1. All patients had nystagmus and foveal hypoplasia. Iris transillumination was assessed using a 5 point grading scale [30]. Consistent with her mild pigmentation defect, the patient with HPS-3 had an iris transillumination score of 0. However, although the children with OCA1 generally had more severe hypopigmentation of their skin and hair compared to those with HPS, iris transillumination scores in children with OCA1 (mean score = 2.8) and HPS (mean score = 2) did not differ significantly (*p* = 0.46).

**Table 3:** Eye Manifestations of Chinese Children with Hermansky-Pudlak Syndrome or Non-syndromic Oculocutaneous Albinism Type 1

Patient	Diagnosis	Visual Acuity OD/OS	Nystagmus	Foveal Hypoplasia	Iris Transillumination Score
215	HPS-1	20/400 20/400	yes	yes	4
351	HPS-3	20/160+ 20/125	yes	yes	0
435	HPS-4	20/150 20/80	yes	yes	3
471	HPS-1	20/200 20/200	yes	yes	1
468	OCA1	20/400 20/640	yes	yes	3
469	OCA1	20/400 20/320	yes	yes	2
470	OCA1	20/200 20/250	yes	yes	3
474	OCA1	20/125 20/125	yes	yes	3

### Platelet Electron microscopy and function

**Fig. 5:**
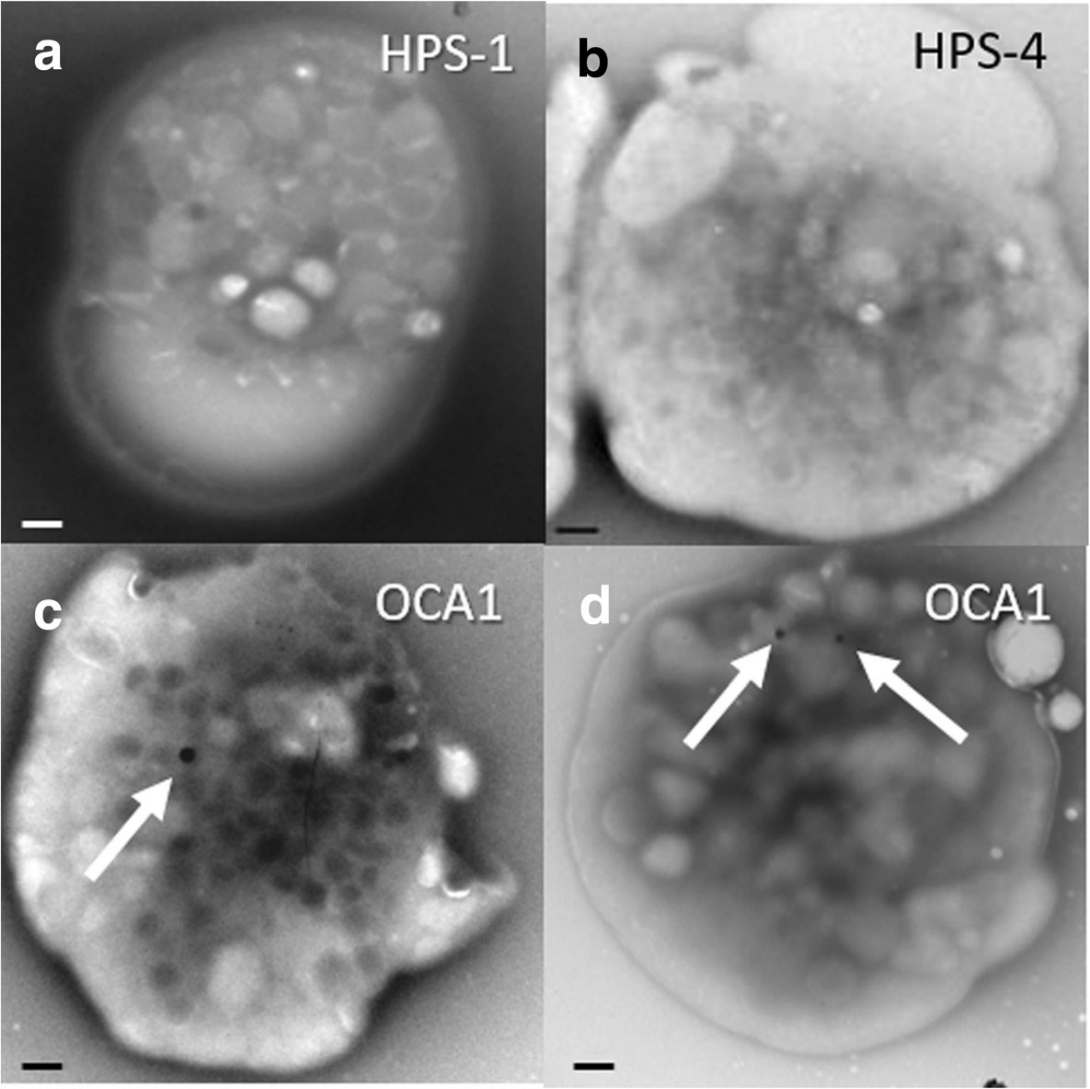
Platelet Imaging in Chinese Children with Hermansky-Pudlak Syndrome or Non-syndromic Oculocutaneous Albinism Type 1. Representative whole-mount transmission electron microscopy images of platelets demonstrate absent dense granules in platelets from Chinese pediatric patients with HPS-1 (**a**) or HPS-4 (**b**). A few dense granules (white arrows) are shown in platelets from two different children with OCA1 (**c**, **d**) (scale bar = 500 nm)

Prothrombin time, activated partial thromboplastin time, and platelet counts were within normal range for patients with HPS or OCA1, except for one patient with HPS with a mildly reduced platelet count (Table 4). Platelet analysis by whole mount transmission electron microscopy showed absent dense granules in the patients with HPS regardless of their HPS subtype (Fig. 5a and b; Table 4). Chinese pediatric patients with OCA1 had 1.35 to 1.9 mean dense granules per platelet (Fig. 5c and d; Table 4). The lower limit of normal for children is 2 dense granules per platelet [31], however, the normal range for children of Chinese heritage is not reported.

**Table 4:** Hematological Profile of Chinese Children with Hermansky-Pudlak Syndrome or Non-syndromic Oculocutaneous Albinism Type 1

Patient	PT [11.6–15.2 s]	aPTT [25.3–37.3 s]	Platelet count [189–394 K/uL]	Mean dense bodies/platelet
215	N/A	N/A	324	0
351	13.6	34.5	160	0
435	13.7	32.7	264	0
471	13.8	34.9	289	0
468	13.9	38.4	268	1.4
469	13.5	32.4	216	1.9
470	14.5	35.3	292	1.9
474	14	37.1	321	1.35

*N/A*, not available

Functional studies of platelets from patients with OCA1 were performed. Platelet aggregation using epinephrine was assessed in whole blood and platelet-rich plasma specimens. Whole blood aggregation studies were normal. However, the secondary wave of platelet aggregation was absent or delayed in response to high or low epinephrine in two patients (468 and 470) with OCA1, and adenosine triphosphate release was absent or delayed in all four patients with OCA1 (Table 5).

**Table 5:** Platelet Aggregation Studies of Chinese Children with Non-syndromic Oculocutaneous Albinism Type 1

Patient	PRP epinephrine low	PRP epinephrine high	PRP epinephrine low ATP release	PRP epinephrine high ATP release
468	absent secondary wave	absent secondary wave	absent	absent
469	normal	normal	delayed	delayed
470	absent secondary wave	delayed secondary wave	absent	absent
474	normal	normal	delayed	delayed

*aPTT*, activated partial thromboplastin time

*ATP*, adenosine triphosphate

*PRP*, platelet rich plasma

*PT*, prothrombin time

## Discussion

Hermansky-Pudlak syndrome and non-syndromic oculocutaneous albinism are inherited disorders that present with pigmentation defects affecting the hair, skin, and eyes. A bleeding diathesis secondary to a platelet storage pool defect, due to absent dense granules, is found in patients with HPS, but has not been reported in OCA1. Notably, bruises in patients with pigment defects are generally easily detected and may be misinterpreted as a bleeding diathesis.

Given some of their shared manifestations, establishing a diagnosis of HPS or OCA1 solely based on history and physical examination is unreliable, but certain clinical findings may raise the suspicion of one diagnosis over the other. For example, striking pigment defects with seemingly no hair color and blue irides were found in our patients with OCA1 compared to less severe hypopigmentation in our patients with HPS. However, pigment defects in HPS vary, and some patients with HPS can have profound hypopigmentation [21, 32]. In addition, colitis resembling Crohn’s disease, neutropenia, immunodeficiency, and pulmonary fibrosis can affect some patients with HPS, and the diagnosis of any of these co-morbidities in a patient with oculocutaneous albinism should raise the index of suspicion for HPS. Pulmonary fibrosis affecting patients with HPS-1, HPS-2 and HPS-4 is an important diagnosis, because it is a major cause of morbidity and mortality in patients with HPS [9, 15]. Medical therapy is not currently approved as treatment for this complication. Anecdotal cases of patients with HPS pulmonary fibrosis receiving long-term open-label pirfenidone suggest that some may benefit from pirfenidone [33]. However, results of clinical trials investigating pirfenidone as treatment for HPS pulmonary fibrosis were inconclusive [34, 35]. Some patients with severe HPS pulmonary fibrosis received lung transplants [36, 37]. Unfortunately, not all lung transplant candidates with HPS-1 receive donor organs, and alloimmunization is a barrier to lung transplantation for some HPS-1 patients [36]; this can be avoided by judicious use of platelet transfusions throughout patients’ lives. Thus, establishing a reliable diagnosis of HPS, identifying the HPS genetic subtype, and long-term clinical planning to maintain candidacy for potential lung transplantation are essential for these patients.

Diagnostic testing of patients presenting with albinism and easy bruising can include platelet analyses and genetic studies. Functional platelet testing can provide results suggestive of storage pool disorders, such as HPS. However, these results are not diagnostic and can be inconclusive, as shown in our patients with OCA1 who had abnormal platelet aggregation test results [38–41]. Indeed, the two patients with OCA1 and a mean of 1.9 dense granules per platelet had differing aggregation studies using platelet rich plasma. We acknowledge that normal ranges of platelet function assays and concentrations of dense granules, which can affect platelet aggregation results, have not been elucidated in Chinese children. The paucity of information in this specific population of patients further complicates the interpretation of their test results. Studies are indicated to define normal ranges in this population and to determine whether or not an unrecognized bleeding diathesis is associated with OCA1. Whole mount transmission electron microscopy of platelets showing absent dense granules is another diagnostic test for patients with HPS [41]. However, proper collection and processing of blood samples are needed to avoid platelet activation and degranulation [42]. In addition, absent dense granules are typically found in all patients with HPS, so this test does not provide insights into HPS genetic subtypes. Indeed, this study showed absent platelet dense granules in patients with HPS-1, HPS-3, and HPS-4, who have differing prognoses. Given the prognostic implications associated with certain HPS genetic subtypes, clinicians should consider evaluating children with oculocutaneous albinism and an increased tendency to bleed using genetic testing for genes associated with HPS, non-syndromic oculocutaneous albinism, Chediak-Higashi syndrome, or Griscelli syndrome. Such genetic testing could be used on a case-by-case basis as a substitute for classical evaluations involving platelet functional analyses or whole mount transmission electron microscopy.

In this cohort of eight Chinese children with clinical features consistent with HPS, genetic sequencing showed that two had HPS-1, one had HPS-3, one had HPS-4, and four had OCA1. Previous studies focusing on Chinese patients with hypopigmentation identified several individuals with OCA1, OCA2, and different HPS subtypes, including HPS-1, HPS-3, HPS-5, and HPS-6 [5, 23, 24, 43]. Notably, our study is the first to report a Chinese patient with HPS-4; this result is consistent with the absence of available data for this novel *HPS4* variant in the East Asian population in ExAC or GnomAD. In addition, some children in our study have unreported genetic variants in *HPS1* and *TYR*, which are predicted to be pathogenic.

## Conclusions

Genetic testing in cases of suspected HPS and OCA is recommended to definitively diagnose patients. Although platelet whole mount transmission electron microscopy and aggregation studies can assist in establishing a diagnosis, the results may be inconclusive and, unlike molecular testing, they do not determine HPS genetic subtypes. Furthermore, these cases illustrate that various HPS subtypes associated with differing prognoses can affect individuals of Chinese ancestry. Our cohort also highlights the genetic heterogeneity of Chinese patients with oculocutaneous albinism and easy bruisability.

## Methods

### Informed consent

Patients’ parents provided written informed consent to participate in protocols 76-HG-0238 (Clinical Trials number NCT00369421; Diagnosis and Treatment of Patients with Inborn Errors of Metabolism or Other Genetic Disorders), and/or 95-HG-0193 (Clinical Trials number NCT00001456; Clinical and Basic Investigations into Hermansky-Pudlak Syndrome), which were approved by the institutional review board of the National Human Genome Research Institute. Testing was performed for diagnostic evaluation of each patient at the National Institutes of Health Clinical Center in Bethesda, Maryland.

### Clinical examinations

Patients were examined for bruising, petechiae, and lung disease. Ophthalmologists with expertise in oculocutaneous albinism also evaluated patients for visual acuity, iris transillumination, nystagmus, foveal hypoplasia and optical coherence tomography (OCT) when possible. Iris transillumination was graded using a 5-point scale as described (grade 0 = no iris transillumination; grade 4 = complete iris transillumination) [30].

Chemistries, complete blood counts, and coagulation tests were performed. Computed tomography scans of the chest with high resolution images were obtained in some patients as described [44].

### Platelet testing

For functional assays, platelets isolated from platelet-rich plasma were subjected to light-transmission aggregometry and adenosine triphosphate release using high and low concentrations of epinephrine as described [45]. For platelet dense granule analysis, transmission electron microscopy of whole mount platelets was performed by an experienced hematologist [46]. For each case, 100–200 platelets were counted.

### Genetic analysis

For patients 215 and 351, genomic DNA isolated from peripheral blood was analyzed by di-deoxy Sanger sequencing for candidate genes. Sequencing of polymerase chain reaction amplification products was performed using a genetic analyzer (Applied Biosystems 3130×; Foster City, CA) as described [46]. For the 6 other patients, genomic DNA isolated from peripheral blood was analyzed by single-nucleotide polymorphism array using the HumanOmniExpress DNA Analysis BeadChip (Illumina, San Diego, CA) and the GenomeStudio software (Illumina) as described [46]. Whole exome sequencing was performed by the National Institutes of Health Intramural Sequencing Center using the HiSeq2000 (Illumina) and the Illumina Genome Analyzer Pipeline software (V.1.13.48.0) as described [46]. Variant filtering was based on allele frequency less than 0.01 with no reported healthy homozygotes in publicly available databases, dbSNP, ExAC, gnomAD and 1000G. Pathogenicity of the identified missense variants or in-frame indels was assessed using online prediction tools, Polyphen, CADD, SIFT, and Mutation Taster. Denovo variants were not identified, because all of the affected individuals were adopted. Compound heterozygosity of the variants were predicted if more than one possible pathogenic variants were observed for a gene. Sanger sequencing in a CLIA (Clinical Laboratory Improvement Amendments) approved laboratory confirmed the identified variants in each patient.

## Abbreviations

CLIA: Clinical laboratory improvement amendments
HPS: Hermansky-Pudlak syndrome
OCA: Oculocutaneous albinism
OCT: Optical coherence tomography

## References

[1] Grønskov K, Ek J, Brondum-Nielsen K (2007). Oculocutaneous albinism. Orphanet J Rare Dis.

[2] Huizing M, Helip-Wooley A, Westbroek W, Gunay-Aygun M, Gahl WA (2008). Disorders of lysosome-related organelle biogenesis: clinical and molecular genetics. Annu Rev Genomics Hum Genet.

[3] Huizing M, Malicdan MCV, Gochuico BR, Gahl WA, Adam MP, Ardinger HH, Pagon RA (2017). Hermansky-Pudlak Syndrome. GeneReviews.

[4] Simeonov DR, Wang X, Wang C, Sergeev Y, Dolinska M, Bower M (2013). DNA variations in oculocutaneous albinism: an updated mutation list and current outstanding issues in molecular diagnostics. Hum Mut..

[5] Wang Y, Wang Z, Chen M, Fan N, Yang J, Liu L (2015). Mutational analysis of the TYR and OCA2 genes in four Chinese families with oculocutaneous albinism. PLoS One.

[6] Introne WJ, Westbroek W, Cullinane AR, Groden CA, Bhambhani V, Golas GA (2016). Neurologic involvement in patients with atypical Chediak-Higashi disease. Neurol.

[7] Introne WJ, Westbroek W, Golas GA, Adams D, Adam MP, Ardinger HH, Pagon RA (2015). Chediak-Higashi syndrome. GeneReviews.

[8] Wei ML (2006). Hermansky–Pudlak syndrome: a disease of protein trafficking and organelle function. Pigment Cell Melanoma Res.

[9] Gahl WA, Brantly M, Kaiser-Kupfer MI, Iwata F, Hazelwood S, Shotelersuk V (1998). Genetic defects and clinical characteristics of patients with a form of oculocutaneous albinism (Hermansky–Pudlak syndrome). New Engl J Med.

[10] Gil-Krzewska A, Murakami Y, Peruzzi G, O'Brien KJ, Merideth MA, Cullinane AR (2017). Natural killer cell activity and dysfunction in Hermansky-Pudlak syndrome. Brit J Haematol.

[11] Gochuico BR, Huizing M, Golas GA, Scher CD, Tsokos M, Denver SD (2012). Interstitial lung disease and pulmonary fibrosis in Hermansky-Pudlak syndrome type 2, an adaptor protein-3 complex disease. Mol Med.

[12] Hengst M, Naehrlich L, Mahavadi P, Grosse-Onnebrink J, Terheggen-Lagro S, Skanke LH (2018). Hermansky-Pudlak syndrome type 2 manifests with fibrosing lung disease early in childhood. Orphanet J Rare Dis..

[13] Huizing M, Scher CD, Strovel E, Fitzpatrick DL, Hartnell LM, Anikster Y (2002). Nonsense mutations in ADTB3A cause complete deficiency of the β3A subunit of adaptor complex-3 and severe Hermansky-Pudlak syndrome type 2. Ped Res.

[14] Anderson PD, Huizing M, Claassen DA, White J, Gahl WA (2003). Hermansky-Pudlak syndrome type 4 (HPS-4): clinical and molecular characteristics. Hum Genet.

[15] Brantly M, Avila NA, Shotelersuk V, Lucero C, Huizing M, Gahl WA (2000). Pulmonary function and high-resolution CT findings in patients with an inherited form of pulmonary fibrosis, Hermansky-Pudlak syndrome, due to mutations in HPS-1. Chest.

[16] Gronskov K, Brøndum-Nielsen K, Lorenz B, Preising MN. Clinical utility gene card for: Oculocutaneous albinism. Eur J Hum Genet. 2014;22. 10.1038/ejhg.2013.307 PMC4350605 24518832

[17] Lewis RA, Adam MP, Ardinger HH, Pagon RA (2013). Oculocutaneous Albinism Type 1. GeneReviews.

[18] Orphanet (2010). Hermansky-Pudlak syndrome.

[19] Anikster Y, Huizing M, White J, Shevchenko YO, Fitzpatrick DL, Touchman JW (2001). Mutation of a new gene causes a unique form of Hermansky–Pudlak syndrome in a genetic isolate of Central Puerto Rico. Nat Genet.

[20] Witkop CJ, Almadovar C, Pineiro B, Babcock MN (1990). Hermansky-Pudlak syndrome (HPS): an epidemiologic study. Ophthal Paediat Genet.

[21] Santiago Borrero PJ, Rodríguez-Pérez Y, Renta JY, Izquierdo NJ, del Fierro L, Munoz D (2006). Genetic testing for oculocutaneous albinism type 1 and 2 and Hermansky–Pudlak syndrome type 1 and 3 mutations in Puerto Rico. J Investig Dermatol.

[22] Tsai CH, Tsai FJ, Wu JY, Lin SP, Chang JG, Yang CF (1999). Insertion/deletion mutations of type I oculocutaneous albinism in chinese patients from Taiwan. Hum Mut.

[23] Wei A, Yang X, Lian S, Li W (2011). Implementation of an optimized strategy for genetic testing of the Chinese patients with oculocutaneous albinism. J Dermatol Sci.

[24] Wei A, Yuan Y, Bai D, Ma J, Hao Z, Zhang Y (2016). NGS-based 100-gene panel of hypopigmentation identifies mutations in Chinese Hermansky-Pudlak syndrome patients. Pigment Cell Melanoma Res..

[25] Sun W, Shen Y, Shan S, Han L, Li Y, Zhou Z (2018). Identification of TYR mutations in patients with oculocutaneous albinism. Mol Med Reports.

[26] Oh J, Ho L, Ala-Mello S, Amato D, Armstrong L, Bellucci S (1998). Mutation analysis of patients with Hermansky–Pudlak syndrome: a frameshift hot spot in the HPS gene and apparent locus heterogeneity. Am J Hum Genet.

[27] Hermos CR, Huizing M, Kaiser-Kupfer M, Gahl WA (2002). Hermansky-Pudlak syndrome type 1: gene organization, new mutations, and clinical/molecular review of non-Puerto Rican cases. Hum Mut. Mutations in brief #568.

[28] Wang Y, Guo X, Li W, Lian S (2009). Four novel mutations of TYR gene in Chinese OCA1 patients. J Dermatol Sci.

[29] Chaki M, Sengupta M, Mukhopadhyay A, Subba Rao I, Majumder PP, Das M (2006). OCA1 in different ethnic groups of India is primarily due to founder mutations in the tyrosinase gene. Ann Hum Genet.

[30] Summers CG, Knobloch WH, Witkop CJ, King RA (1988). Hermansky-Pudlak syndrome: ophthalmic findings. Ophthalmol.

[31] Sorokin V, Alkhoury R, Al-Rawabdeh S, Houston RH, Thornton D, Kerlin B (2016). Reference range of platelet delta granules in the pediatric age group: An ultrastructural study of platelet whole mount preparations from healthy volunteers. Ped Develop Pathol..

[32] Witkop C (1979). Albinism: hematologic-storage disease, susceptibility to skin cancer, and optic neuronal defects shared in all types of oculocutaneous and ocular albinism. The Alabama J Med Sci.

[33] O'Brien KJ, Introne WJ, Akal O, Akal T, Barbu A, McGowan MP, Merideth MA, Seward SL, Gahl WA, Gochuico BR (2018). Prolonged treatment with open-label pirfenidone in Hermansky-Pudlak syndrome pulmonary fibrosis. Mol Genet Metab.

[34] Gahl WA, Brantly M, Troendle J, Avila NA, Padua A, Montalvo C (2002). Effect of pirfenidone on the pulmonary fibrosis of Hermansky-Pudlak syndrome. Mol Genet Metabol..

[35] O'Brien K, Troendle J, Gochuico BR, Markello TC, Salas J, Cardona H (2011). Pirfenidone for the treatment of Hermansky-Pudlak syndrome pulmonary fibrosis. Mol Genet Metabol.

[36] El-Chemaly S, O’Brien KJ, Nathan SD, Weinhouse GL, Goldberg HJ, Connors JM (2018). Clinical management and outcomes of patients with Hermansky-Pudlak syndrome pulmonary fibrosis evaluated for lung transplantation. PLoS One.

[37] Lederer DJ, Kawut SM, Sonett JR, Vakiani E, Seward SL, White JG (2005). Successful bilateral lung transplantation for pulmonary fibrosis associated with the Hermansky-Pudlak syndrome. J Heart Lung Transplant.

[38] Miller CH (2013). Laboratory diagnosis of platelet function defects. Transfusion Med Hemost.

[39] Pai M, Wang G, Moffat KA, Liu Y, Seecharan J, Webert K (2011). Diagnostic usefulness of a lumi-aggregometer adenosine triphosphate release assay for the assessment of platelet function disorders. Am J Clin Pathol.

[40] Schinella RA, Greco MA, Garay SM, Lackner H, Wolman SR, Fazzini EP (1985). Hermansky-Pudlak syndrome: a clinicopathologic study. Hum Pathol.

[41] Witkop CJ, Krumwiede M, Sedano H, White JG (1987). Reliability of absent platelet dense bodies as a diagnostic criterion for Hermansky-Pudlak syndrome. Am J Hematol.

[42] Magnette A, Chatelain M, Chatelain B, Ten Cate H, Mullier F (2016). Pre-analytical issues in the haemostasis laboratory: guidance for the clinical laboratories. Thromb J.

[43] Wei A, Lian S, Wang L, Li W (2009). The first case report of a Chinese Hermansky-Pudlak syndrome patient with a novel mutation on HPS1 gene. J Dermatol Sci.

[44] Rouhani FN, Brantly ML, Markello TC, Helip-Wooley A, O'brien K, Hess R (2009). Alveolar macrophage dysregulation in Hermansky-Pudlak syndrome type 1. Am J Respir Crit Care Med.

[45] O'Brien KJ, Lozier J, Cullinane AR, Osorio B, Nghiem K, Speransky V (2016). Identification of a novel mutation in HPS6 in a patient with hemophilia B and oculocutaneous albinism. Mol Genet Metabol..

[46] Bryan MM, Tolman NJ, Simon KL, Huizing M, Hufnagel RB, Brooks BP (2017). Clinical and molecular phenotyping of a child with Hermansky-Pudlak syndrome-7, an uncommon genetic type of HPS. Mol Genet Metabol..

